# Beverage intake and obesity in Australian children

**DOI:** 10.1186/1743-7075-8-87

**Published:** 2011-12-12

**Authors:** Peter M Clifton, Lily Chan, Chelsea L Moss, Michelle D Miller, Lynne Cobiac

**Affiliations:** 1 Nutritional Interventions Laboratory, Baker IDI Heart and Diabetes Institute, 75 Commercial Rd, Melbourne, 3004, Australia; 2 Department of Medicine, School of Medicine, University of Adelaide, Adelaide 5000, Australia; 3 Department of Nutrition and Dietetics, Faculty of Health Sciences, Flinders University, Bedford Park, South Australia 5042 Australia

**Keywords:** beverage, sugar sweetened, cross sectional survey, children

## Abstract

**Background:**

There have been increases in the obesity and overweight rates in Australian children over the past 25 years and it has been suggested that sugar sweetened beverages (SSB) have played a role in this increase.

**Objective:**

The objectives of this study were to: (1) examine SSB intakes in the 2007 Australian Children's Nutrition and Physical Activity Survey (2) relate SSB intake to rates of overweight and obesity, socio-economic status (SES), TV viewing time, and activity levels and (3) compare 2007 SSB intakes with data from the 1995 National Nutrition Survey.

**Design:**

A computer assisted 24 h dietary recall in 4,400 children aged 2-16 years was performed.

**Results:**

In the 2007 survey 47% of all children reported drinking SSBs with 25% consuming sugar sweetened soft drinks on the day of the survey. The mean consumption of soft drink was 436 g/d/consumer. Activity levels were unrelated to SSB consumption. Television viewing was positively related to soft drink consumption with a difference of 55 g/day from bottom to top tertile of time spent TV viewing (p = 0.015) in children aged 9-16 years. 55% of SSB consumption occurred at home and 10% occurred at school. Lower SES status was associated with a greater prevalence of SSB consumption- 30% for the lowest SES quartile vs 19% in the highest quartile. The proportion of overweight who consumed SSBs (which excludes 100% fruit) was not different from the non-overweight children although the proportion of SSB consumers in the 6% of children who were obese was significant compared with the non-overweight children (59% vs 47%, p < 0.05). In the 2007 survey 23% of children were overweight (17%) or obese (6%) while in the 1995 survey this figure was 21%. The proportion of children consuming SSBs in 1995 and 2007 for selected age groups were: 2-3 years - 25.8% and 12.8% respectively and 4-7 years - 33.6% and 20.5% respectively (p < 0.001 for both).

**Conclusions:**

This cross-sectional data set provides evidence that SSB consumption for Australian children is still high despite the decrease since 1995 in some age groups. It provides little support to conclude that overweight in children is currently being driven by excessive SSB consumption although it may be factor in some obese children. Conclusions are limited by the cross sectional nature of the study.

## Introduction

In the USA increases in sweetened beverage consumption have been associated with the increase in adult and childhood obesity [1,2] and calls have been made for increases in taxation on these products to reduce consumption [3,4]. The use of high fructose corn syrup has also been associated with the increase in obesity [5], although some authors have disputed that this is a causal relationship [6,7]. The rate of obesity and overweight in children in Australia is now approximately 25% [National health screening 2007/2008, [8]] but there has been little exploration of the relationship between soft drink consumption and body weight in Australia apart from one small study [9,10]. However Australian authors have also proposed the weight of evidence indicates that soft drinks are a major issue in childhood and adolescent obesity and that the doubling of consumption of soft drinks from 47 L/head [adult and children] in 1969 to 113 L/head in 1999 is related to increases in childhood obesity [11]. We utilised information from the 2007 Australian National Children's Nutrition and Physical Activity Survey to examine this issue and made comparisons with data in children from the 1995 National Nutrition Survey, Australian manufacturers' sales data and with other small surveys in children.

## Subjects and Methods

The 2007 Australian National Children's Nutrition and Physical activity survey gathered information on 4,487 children aged 2-16 years between February and August 2007 [12]. The survey measured dietary intakes of food and beverages, use of supplements during the previous 24 hours, some food habits, height, weight and BMI, waist circumference, and demographic characteristics in all children and time spent in physical activity and screen time and the number of steps taken daily in the 9-16 and 5-16 year olds respectively. Households with children were randomly selected using random digit dialling from all Australian states and territories in metropolitan, rural and remote areas. The survey design was a 2-stage cluster design with stratification. The primary sampling units were postcodes. The number of children included from each state was proportional to the population of children in that state or territory. The response rate of eligible children was 41%. The data were collected at a face-to-face home visit with computer assistance. Questions were specifically asked about beverages on 2 occasions during the review of food and beverages in the previous 24 h. Physical activity was measured using two different approaches. Use of time was measured in children aged 9-16 years using a validated computerised 24-hour recall [13]. Children recalled a total of four days. Pedometers were also used to measure the average number of steps taken daily over 6 days by children aged 5-16 years. There was quota sampling with 500 boys and 500 girls in each age group [2-3, 4-8, 9-13 and 14-16 years] recruited nationally with an additional 400 in South Australia across all age groups. The sample appeared to collect fewer single parent families and have a higher family income than a nationally representative sample.

Personal interview data from the face to face dietary 24-hour recall interview only was used for this report. "Consumers" of individual non-dairy non-alcoholic beverage items were defined as any children who had consumed that particular non-dairy non-alcoholic beverage item in any amount on the day of the 24 hour food recall collected during the computer assisted Data in the 2007 survey were not gathered during the summer.

BMI was assessed using measured weight and height and the cut points for overweight and obesity were those described by Cole et al [14,15] from the International Obesity Taskforce which have been used extensively in Australia since their publication. Socio economic status of the child was based on one of the Socio-Economic Indexes for Areas [SEIFA], Australia - Index of Relative Socio-economic Disadvantage [IRSD], which focuses primarily on disadvantage, and is derived from Census variables like low income, low educational attainment, unemployment, and dwellings without motor vehicles [16].

Data from the 1995 National Nutrition Survey are available online from the Australian Bureau of Statistics [17,18] while weight data from this and previous 1985 surveys have been reanalysed by Magarey et al [19]. The 1995 Nutrition survey was part of a National Health Survey of preselected households in which a proportion of participants were asked face to face if they wished to participate in a nutrition survey. The survey was a multiple pass 24 h diet recall with a confirmation survey in 10% of participants. The nutrition survey was conducted in all months of the year.

Manufacturers data on the total annual sales of sugar sweetened and non nutritive sweetened beverages was obtained directly from Coca Cola South Pacific.

### Statistics

All estimates [means, medians and percentages] were population weighted to take account of the non-proportionate sampling survey procedure. Weightings in this survey were based on age, gender and region [by State/Territory and by Capital City/Rest of State]. Statistical analyses were performed using Stata Version 10.1 [StataCorp, Texas, USA]. Jack knife replicate weights were used to allow for the complex survey design. Pearson Chi-Square tests were used to determine differences between categorical variables. Linear regression was used to test the association between dietary intakes and independent variables [ie. food group consumption and television viewing].

The p value of all results has been Bonferroni adjusted to take into account the 5 major endpoints in this study - activity, TV viewing, body weight, SES categories and comparison with 1995 intakes.

The major focus of this paper will be on sugar sweetened, non dairy, non alcoholic beverages [called sugar sweetened beverages or SSBs in other publications]. This includes sugar sweetened carbonated drinks [soft drinks in Australia or soda in the USA], fruit drinks, cordials, flavoured waters, energy drinks, iced tea and sports drinks and excludes 100% fruit juice. Water intake will not be addressed in detail.

## Results

### Consumption of sweetened beverages

**Figure 1:**
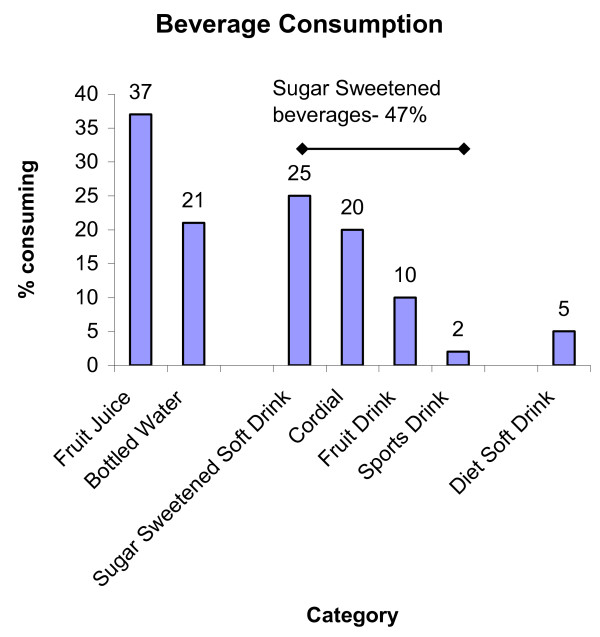
**Categories of beverage consumed by children of all ages (n = 4487)**.

Forty-seven percent of children consumed sugar sweetened beverages and 7% consumed non nutritive sweetened beverages. Twenty-five percent of children drank sugar sweetened soft drink on the day of the survey with a mean intake in consumers [only] of 436 g/day and a mean intake of 127 g/day across all children in the survey [including consumers and non-consumers]. 37% of children in this study consumed 100% fruit juice [Figure 1]. Overall 21% of children aged 2-16 years reported that they drank bottled water and 68% drank tap water on the day of the survey.

### Consumption patterns by age and gender

There was an increase [P < 0.01] in the number of consumers of sugar sweetened soft drinks increasing from 9.7% in the 2-3 year age group to 32.9% in the 14-16 year age group with no differences in the trend between boys and girls. A similar trend was seen in non-nutritive soft drinks increasing from 2.2 to 8.6% across this age range [P < 0.01]. Similar trends were seen in sports and energy drinks although the numbers of children who consumed these drinks was relatively low [data not shown]. Overall 4.7% more boys consumed sugar sweetened soft drinks than girls [P = 0.01]. In consumers aged 9-13 years the 3^rd ^tertile of intake of sugar-sweetened beverages was > 600 g/d while in the children aged 14-16 years the 3^rd ^tertile was > 721 g/d.

Table 1 shows the major categories of beverages consumed.

**Table 1:** Percentage of children consuming major categories of non alcoholic, non dairy beverages and the mean consumption [g/d/consumer] in consumers.

%					Mean g/d per consumer			
	**Age group**				**Age group**			
Boys	2-3	4-8	9-13	14-16	2-3	4-8	9-13	14-16
Fruit juice	41	42	34	35	214 ± 168	295 ± 218	347 ± 219	406 ± 265
Fruit Drinks	8	12	9	8	232 ± 137	267 ± 134	311 ± 181	473 ± 517
Cordial	23	21	22	21	401 ± 383	377 ± 246	471 ± 346	600 ± 542
Sugar sweetened soft drinks	10	19	34	38	200 ± 112	335 ± 185	496 ± 296	591 ± 349
Diet soft drinks	2	4	6	10	197 ± 137*	242 ± 125	392 ± 206	489 ± 256
								
Girls								
Fruit juice	37	35	36	39	213 ± 157	243 ± 158	299 ± 201	341 ± 229
Fruit Drinks	9	13	11	9	260 ± 182	269 ± 180	275 ± 136	311 ± 139
Cordial	18	20	20	17	319 ± 200	348 ± 257	436 ± 330	470 ± 338
Sugar sweetened Soft drinks	19	17	29	28	214 ± 143	279 ± 206	407 ± 266	507 ± 328
Diet soft drinks	3	4	6	8	133 ± 122*	223 ± 98	378 ± 254	417 ± 206

1. One day food intake data collected via 24 hour recall at personal interview; population weights applied; n = 1682 for fruit juice; n = 431 for fruit drink; n = 918 for cordial; n = 1046 for sugar sweetened soft drinks; n = 238 for diet soft drinks; n = 4433 for all non-dairy non-alcoholic beverages;

2. Estimates calculated based on sample size of 20 or less for each group are marked with an asterisk

### Association with weight status

**Figure 2:**
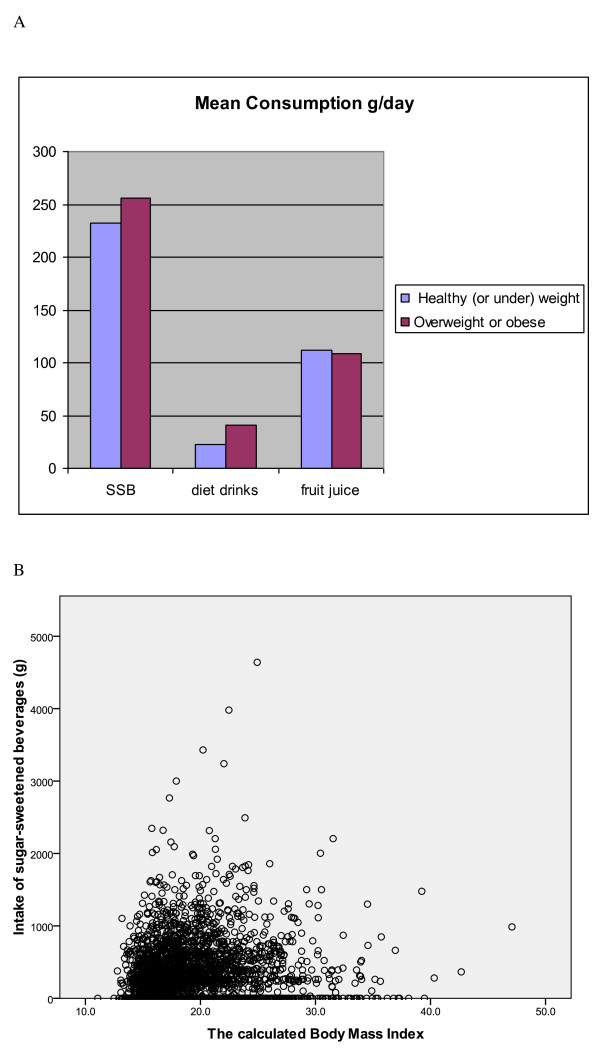
**(a)** Mean consumption of beverages across all children (consumers and non consumers). **(b)** Consumption of SSB across all children plotted against BMI.

A higher proportion of overweight or obese children consumed sugar sweetened beverages than those of normal and underweight [50% vs 47% unadjusted P = 0.046] but this difference was not significant after Bonferroni adjustment [Table 2]. If only the obese children are considered and compared to non-over weight children the difference in proportion is statistically significant after Bonferroni correction [59% vs 47%, p = 0.03]. Figures 2a shows mean intakes per head across the whole population and Figure 2b individual values of SSB intake. Mean intakes in consumers were not different comparing overweight and obese children with normal and underweight children [511 vs 499 g/d/consumer]. Ten percent of overweight or obese children consumed non nutritive sweetened beverages compared with 6% of normal weight children [P < 0.01] but mean intakes per consumer were similar [402 vs 378 g/d].

**Table 2:** Association between weight status and beverage consumption

	Healthy/underweight				Overweight/obese			
	**2-3 years**	**4-8 years**	**9-13 years**	**14-16 years**	**2-3 years**	**4-8 years**	**9-13 years**	**14-16 years**
SSB								
% consuming	31	42	55	52	40	45	56	52
Mean daily intake per consumer [g]	339 ± 308	379 ± 257	545 ± 387	649 ± 465	345 ± 277	422 ± 274	527 ± 351	646 ± 516
Non Nutritive SSB								
% consuming	4	5	7	9	3	9	11	15
Mean daily intake per consumer [g]	214 ± 149	290 ± 207	418 ± 274	460 ± 294	230 ± 114	253 ± 140	388 ± 296	554 ± 261
Fruit Juices								
% consuming	41	38	37	37	32	39	32	36
Mean daily intake per consumer [g]	215 ± 163	270 ± 199	322 ± 219	365 ± 240	206 ± 159	281 ± 182	323 ± 188	400 ± 276

1. One day food intake data collected via 24 hour recall at personal interview; population weights applied; n = 3477 for healthy/underweight; n = 1010 for overweight/obese; n = 1325 for healthy/underweight juice consumers; n = 357 for overweight/obese juice consumers; n = 1579 for healthy/underweight sugar sweetened beverage consumers; n = 492 for overweight/obese sugar sweetened beverage consumers; n = 210 for healthy/underweight non nutritive sweetened beverage consumers; n = 104 for overweight/obese non nutritive sweetened beverage consumers;

2 Weight status classified according to standard definitions published by the International Obesity Task Force [IOTF] [Cole et al. 2000; Cole et al.2007].

3 Includes sugar sweetened soft drinks, cordials, fruit drinks, flavoured waters, energy drinks, iced tea and sports drinks.

4 Includes non nutritive sweetened fruit drinks, soft drinks, cordials, flavoured waters and energy drinks.

### Location of consumption

A large proportion of all beverages [55%-77%] were consumed at home and only about 10% or less was consumed at school, kindergarten or pre school.

### Association with behaviours associated with weight gain

**Figure 3:**
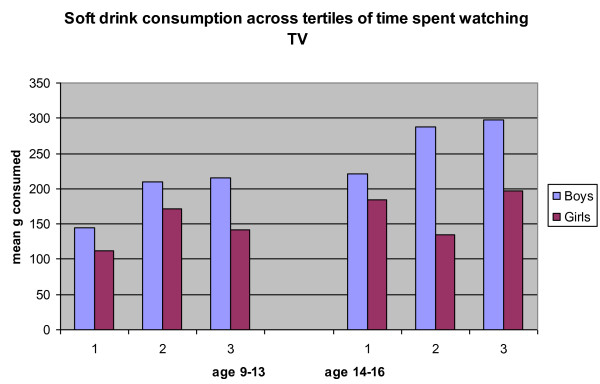
**Soft drink consumption across tertiles of time spent watching TV**. 1st Tertile 0 to < 106.5 min of TV viewing. 2nd Tertile 106.5 to < 178.5 min of TV viewing. 3rd Tertile 178.5 min or more of TV viewing.

The number of hours spent watching television was computed for those aged 9 years or older. Overall, with increasing amounts of television exposure [from < 107 min to > 179 min], there were significantly higher reported mean intakes of soft drinks [nutritive and non nutritive], increasing from 156 g/d to 210 g/d across tertiles of television exposure in all children aged 9-16 years. Those within the first tertile of television exposure consumed significantly less soft drink than those in the third tertiles [P = 0.01]. This was especially marked in boys [175 g increasing to 250 g][Figure 3].

There was no relationship between soft drink intake and whether the child met or did not meet physical activity guidelines [guidelines are met if child performed at least 60 minutes of moderate to vigorous physical activity level on most days surveyed [i.e. 3 out of 4 days], data collected over 2 × 2 days]. The difference seen across tertiles of TV viewing was the same in active and less active children.

### Percentage energy from soft drinks

The percentage of total energy from sugar sweetened soft drinks was 6.6% in consumers ranging from 4.6% in the youngest age group to 7.5% in the oldest age group. Similar energy intakes were seen in consumers with fruit drinks [6.2%], cordial [4.2%], iced tea [6.7%] flavored water [4.5%], sports drinks [6.5%] and energy drinks [7.1%]. This compares with 5.4% for fruit juice and 5.4% for all non dairy, non alcoholic beverages in consumers. Across all children [ie including consumers and non-consumers] 1.6% of total energy came from sugar sweetened soft drinks, 2% from fruit juice and 0.8% from cordials and 0.6% from fruit drinks.

The mean energy intake of sweetened beverage [including non-nutritive sweetened beverages] consumers was 1129kJ /day, greater than non-consumers across all groups with 31g/day greater mean total sugars intake [both P < 0.01].

### Association with socio economic status

Table 3 shows the association between lower SES status and SSB consumption which is highly significant in all age groups [P < 0.01] and was an absolute 15-16% difference between top and bottom quartiles. In addition a higher proportion of children with lower SES consumed sugar sweetened soft drinks [30.2%] compared with the highest socio-economic group [19.4%, P < 0.01]. This was similar in all age groups, although not statistically significant in the 14-16 year age group, but was somewhat more marked in the 2-3 year old age group [13.6 vs 5%, P < 0.01]. The mean consumption in consumers only of sugar sweetened soft drinks was greater in the two lower SES quartiles [440-467 g/d] while the estimated mean intake was lowest in those children in the top SES quartile [407 g/d]. This was marked in the 14-16 year age group [609 g/d for low SES vs 483 g/d in the highest SES quartile]. The mean consumption of bottled water consistently increased from lowest to highest SES quartile for consumers only [from 646 g/d in lowest quartile to 747 g/d in the highest quartile]. Table 3 also shows the weight status by SES; this showed that the combination of the prevalence of overweight and obesity was not different between SES groups although obesity alone increased from 4% in the highest SES group to 9% in the lowest SES group [p < 0.001]. The difference in SSB consumption rates in the obese group does not account for the overall difference between SES quartiles.

**Table 3:** Relationship between SES and weight status

SES tertile	1	2	3	4
Age group [yr]				
2-3	39	35	37	23
4-8	49	48	41	34
9-13	64	61	50	46
14-16	62	52	51	47
Weight status [%]				
Underweight	5	3	6	5
Normal weight	69	71	74	75
Overweight	17	20	15	16
Obese	9	6	6	4

1. Percentage of the population consuming SSBs in each SES [SEIFA-IRSD] quartile and percentage of the population overweight or obese in each SES quartile

2. One-day food intake data collected via 24-hour recall at personal interview; population weights applied

3. SES as indicated by SEIFA [Index of Relative Socio-Economic Disadvantage 2006] where the 1st quartile represents those who are most disadvantaged and the 4th quartile represents those who are least disadvantaged

4. Weight status classified according to standard definitions published by the International Obesity Task Force [IOTF] [Cole et al. 2000; Cole et al.2007].

### Changes since the previous survey in 1995

Based on the available data there would appear to be a decrease in the proportion of children aged 2-3 years and aged 4-7 years who consumed soft drinks, flavoured mineral waters and electrolyte drinks with a drop from 25.8% to 12.8% in the 2-3 year olds and from 33.6% to 20.5% in the 4-7 year olds [P < 0.01 for both by Chi squared analysis, Table 4]. However the surveys are not directly comparable because of different sampling methods therefore these statistics may be an overestimate of the true difference. In the 1995 National Nutrition Survey sugar sweetened soft drinks [3.3%] cordials [2.7%] and fruit drinks [1.4%] were major contributors to the estimated total energy intake of all children [20]. These values are higher than reported in the 2007 survey where 1.6% of energy came from soft drinks, 2% from fruit juice and 1.4% from cordials and fruit drinks.

**Table 4:** Beverage consumption in 1995 and 2007

Age Range [yr]	% consuming		Grams/consumer [median]	
	**1995**	**2007**	**1995**	**2007**
Boys				
2-3	31	12	197	175
4-7	36	22	321	261
8-11	39	37	393	391
12-15	48	50	522	476
16-18 [only 16 in 2007]	61	54	772	591
				
Girls				
2-3	21	13	209	167
4-7	31	19	261	209
8-11	37	34	387	318
12-15	42	38	391	391
16-18 [only 16 in 2007]	54	40	393	480

1. Comparison between National Nutrition Survey 1995 [n = 3007] and the 2007 Children's Survey [n = 4487].

2. Percentage of children consuming soft drinks, flavoured mineral waters and electrolytes drinks [and ice tea and energy drinks in 2007].

3. One day food intake collected via 24 hour recall at a personal interview. Population weights were applied.

### Relationship to dairy milk consumption

High and low consumers of sugar sweetened soft drinks did not differ in their unflavoured milk consumption [mean of 217 g/day for the lowest tertile vs 223 g/day in the top tertile] although there was a small, non significant difference in milk intake between non consumers and consumers of sweetened beverages [mean of 273 g/day vs 212 g/day]. The mean daily intake in consumers of sweetened beverages was significantly lower for calcium [934 mg vs 965 mg, p = 0.03] when compared to nonconsumers.

### Correlation of survey intake data with beverage manufacturers sales data

Based on the reported consumption data of 25% of children drinking sugar sweetened soft drink with a mean intake in consumers of 436 g/day, the estimated total annual consumption in this age group would be 159 L/head per consumer or 40 L/head overall. The major item in the sugar sweetened beverage category was carbonated soft drinks although 20% reported drinking cordial [mean intake 429 g/day], 10% fruit drinks [mean intake 294 g/day] and 2% sports or energy drinks [mean intake 620 g/day]. This equates to an additional 46 L/head/year with a mean total of 86 L/head per year. These values tally very well with the estimate consumption of 80 L/head/year of sugar sweetened beverages from a total population of 22 million people consuming 1500,000,000 L per year of sweetened beverages. This concordance in intake data suggests that children in the 2007 survey are representative of the whole population and are not a biased sample despite only a 41% response rate.

### Obesity rates in children in Australia 1985-2007

**Figure 4:**
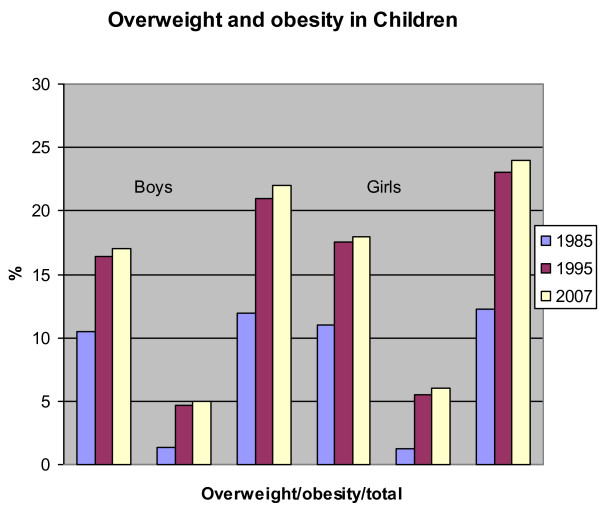
**Rates of overweight and obesity in children aged 2-16 in 1985, 1005 and 2007**. Adapted from Magarey et al [16].

Around 25% of children in Australia are overweight or obese and this has increased from about 5% in the 1960's. From 1985 to 1995 the overweight and obesity rate doubled and the obesity rate tripled [19]. In 1995, 21% of boys and 23% of girls aged 2-17 years were overweight or obese; from 1985 to 1995, the proportion of obese boys aged 7-15 years increased from 1.4% to 4.7%, and the proportion of obese girls in this age group increased from 1.2% to 5.5% [Figure 4]. Between the 1995 and 2007 surveys there were only very small increases with an overweight rate of 17% and an obesity rate of 5% in boys and 18% and 6% in girls in 2007. The highest obesity rates of 7% were seen in boys aged 9-13 years and girls 9-16 years.

The South Australian Child Youth Help Study in 2002 indicated that obese preschoolers aged 4 years rose from 3.5% in girls and 3.2% in boys in 1995 to 5.8% for girls and 4.1% for boys in 2002 [20]. In data from South Australia's preschool health check [ages 4-5 years] the rates of overweight [14%] and obesity [5%] the rates have not changed from 1999 to 2008, following a significant jump from 1995 where the combined rates increased from 11 to 19% [20].

The overweight and obesity rates are slightly greater in the 2007-08 National Health Survey [5000 children aged 0-17 ][21] where 24.9% of children aged 5 - 17 years were overweight or obese. 25.8% of boys and 24.0% of girls were either overweight or obese while the obesity rates were 9.7% in boys and 5.8% in girls.

### Changes in intake as assessed by manufacturers data

Total sugar sweetened beverage consumption in Australia showed a weak 12% trend downwards from 2002-2006 [from about 1600 m litres to 1400 m litres, p = 0.016]. This trend appears to have ceased in the period from 2006 to 2009. However non sugar sweetened beverages [ie diet drinks] have increased significantly [p < 0.001] by 56% and this trend has continued beyond the survey period out to 2009. There have been large relative increases in energy drinks and teas although overall levels of consumption are still low [22 and personal communication-Coca Cola South Pacific ].

## Discussion

The 2007 children's survey, a cross sectional study, showed no relationship between overweight status and sugar sweetened beverage consumption on the day of the survey, although there was a significant difference in consumption patterns in the small group of obese children. Despite small increases in the rates of both overweight and obesity between 1995 and 2007 there was a steady small decline in sugar sweetened soft drink consumption between 2002 to 2006 and an increase in diet drinks based on manufacturers' data which is also reflected by a decline in mean amount consumed of sugar sweetened beverages [as a percentage of energy] from the 1995 to 2007 surveys. Conclusions about the causal relationship between soft drink consumption and overweight are limited by the cross sectional nature of the study. Reverse causation, although possible in the overweight group, is unlikely given the observed positive relationship between SSB consumption and obesity. It would be expected that individuals in the obese group would be more likely to swap diet drinks for SSB given that many in the overweight group may be unaware of their weight status. The overweight/obese group combined consumed more diet drinks than the normal weight group.

However two well established risk factors for obesity, lower SES and higher TV viewing hours were associated with increased sugar sweetened beverage consumption in this survey. Consumption of sugar sweetened soft drinks was nearly twice as common in children from low SES areas with larger volumes consumed as well suggesting this may be a factor in the almost doubled rate of obesity in this group. In contrast sugar sweetened beverages appeared not to be a factor in overweight children regardless of SES quartile and the greater consumption of SSB in the lower SES quartiles could not be explained by the behaviour of the obese group alone. In an analysis of the 1995 National Nutrition Survey no relationship was seen between beverage consumption and SES [23] which suggests that beverage behaviour has changed over the last 12 years.

Two other Australian studies [24,25] have also shown that a higher intake of soft drinks was associated with lower socio-economic status [SES] in school students. Apart from the 2007 Children's Survey and the Barwon area surveys [26] there are little data from Australia on the relationship between BMI and soft drink consumption. Tam et al [9] followed 281 children aged 7-8 years from Western Sydney [a low SES area] for 5 years and found that the intake of soft drink/cordial [as assessed by 3 day weighed food record] was 10 g/day higher in children who were overweight/obese at follow-up compared to those who had an acceptable BMI at both baseline and follow-up [P = 0.002]. Fruit juice/fruit drink and milk consumption were not related to weight status. The relationship between TV viewing and weight status in Australian children is limited but in accord with other studies is relatively weak. In a large Victorian study in a total of 2862 children aged 5-13 years child mean BMI z-scores were significantly related to television viewing [F = 10.23, P < 0.001] but accounted for only 1% of total BMI variance. When parental BMI, parental education, number of siblings, food intake, organized exercise and general activity level were adjusted for as potential confounders, television ceased to be independently significantly related to child BMI. Using adjusted logistic regression, the odds of being overweight and obese generally increased with increasing television viewing [27]. TV viewing for more than 2 hours/day in Melbourne primary school children [n = 1560] is also associated with both high energy drinks and savoury snack consumption [28]. Although the relationship between weight and SSB consumption is not clear the increased consumption of SSBs in those of low SES status and those who watch many hours of TV is likely to increase their risk of type 2 diabetes and metabolic syndrome in later life [29]

In the UK only weak cross sectional relationships between sugar-sweetened beverages and weight status are apparent [30]. In the UK National Dietary and Nutritional Survey of Young People [n = 1,294 aged 7-18 years] non-milk extrinsic sugars and caloric soft drinks [excluding 100% fruit juice] were quantified by their contribution to energy intake. The BMI z-score was weakly inversely correlated with percentage energy from non milk extrinsic sugars after adjustment for under-reporting and dieting [r = -0.06, P = 0.03]. The percentage of energy from soft drinks was not associated with the BMI z-score or physical activity. After excluding under-reporters and dieters, the heaviest children [top quintile of BMI z-scores] consumed more total energy [+1,220 kJ/day] than those in the lowest quintile, but only 60 kJ [5%] was from soft drinks. The top tertile intakes of caloric soft drinks were not associated with overweight [OR = 1.39, CI = 0.96-2.0] but when divided into quintiles obesity rates were higher in the top quintile in which children consumed a -mean of 870 kJ/day or about 550 g/d of soft drink [OR = 1.67, CI = 1.04-2.66, P = 0.03].

In the USA it would appear that a similar proportion of children consume sugar sweetened beverages as in Australia although the rates are lower in Australia in the 2007 survey as the survey period was one day only. In the 1994-6 Continuing Survey of Food Intake [1810 children aged 2-16 years] 51% of pre-schoolers up to 82% of adolescents consumed soft drinks over the 2 days of the survey. This was a 65-74% increase over the previous survey in 1977-79 [31]. In a recent 2010 USDA report [32] the estimated current caloric contribution of sugar sweetened beverages in children 2-19 years was 193 kilocalories per day or about 480 g of drink. There has been a fall in sugar sweetened soda in the USA from 1999 to 2006 of about 30 g/day and a flattening of the increase in overweight rates in children. In NHANES 111 [1988-1994] beverages contributed 20-24% of energy across ages 2-19 years while soft drinks provided 8% of energy in adolescents [33]. Per-capita daily caloric contribution from sugar-sweetened beverages and 100% fruit juice increased from 242 kcal/day to 270 kcal/day in 1999-2004. There was no change in per-capita consumption among white adolescents but significant increases among black and Mexican American youths [34].In a secondary analysis of 1572 preschool children examined in NHANES 1999-2002, O'Connor et al [35] found that 83% of children drank milk, 48% drank 100% fruit juice, 44% drank fruit drink, and 39% drank soda. Weight status of the child had no association with the amount of total beverages, milk, 100% fruit juice, fruit drink, or soda consumed. There was no clinically significant association between the types of milk [percentage of fat] consumed and weight status. Daily total energy intake increased with increased consumption of milk, 100% fruit juice, fruit drinks, and soda, but there was not a statistically significant increase in BMI on the basis of quantity of milk, 100% fruit juice, fruit drink, or soda consumed.

There are few long term prospective studies examining beverage intake and BMI changes and those in the literature mostly have data from before 1998 so they may not reflect recent changes in behaviour. Forshee et al [36] performed a meta analysis of 10 longitudinal and 2 randomized controlled trials and found that BMI changed by 0.004 units for each serving/day change in SSBs which was not significant [95% confidence interval -0.006, 0.014] although Malik reanalysed the same studies and found a positive relationship which was significant in only those studies which did not adjust for total energy with an 0.08 BMI units per change in 12 oz serving of SSB [37]. Part of the reasons for the difference lay in the use by Malik of correct scaling of the original data as well as differing weights of each study in a random effects analysis. However even with these changes the effect on BMI was still not significant The main point of difference was the use or otherwise of results from studies that had adjusted for energy intake as there was no relationship seen in those which adjusted for energy intake whereas there was a clear relationship in those which did not adjust. This suggests that SSBs contribute to the overall increase in energy seen in those who gain weight but do not have a unique out of proportion contribution.

In California there have been substantial declines from 2003 to 2007 in high SSB consumption [defined as having more than one SSB drink the previous day] varying from a reduction of 5% to 37.5% with the greatest change in the 12-17 age group. Obesity rates also declined in the 2-5 and 6-11 age group but not the 12-17 age group. Children and teenagers in 2005 and 2007 were significantly less likely than those surveyed in 2003 to have high SSB consumption after adjusting for gender, age, race/ethnicity, poverty level, and parental education [P < 0.001] [38]. In adults Mozzafarian et al [39] found that an increase in one serve per day of SSB was associated with an increase in body weight by 1lb over 4 years after full adjustment for all other dietary factors. This data was derived from a very large study of nearly 121,000 men and women in the USA who were tracked over a 12-20 year period. The results were very similar across men and younger and older women. Energy intake was not included as a covariate as it could be a direct mediator of the weight gain but as all the more than 20 other dietary factors were added to the regression it would appear that an increase in SSB is not necessarily accompanied by a significant increase in other foods associated with weight gain.

In conclusion the 2007 Children's Survey found there was no cross sectional relationship between overweight and sugar-sweetened beverage consumption although consumption was higher in obese children, lower SES children and children who were in the highest tertile of TV watching; consumers of SSBs consumed more total sugars and total energy than non consumers. Since the last survey in 1995 sugar-sweetened beverage consumption appears to have decreased in some groups and the mean energy contribution from SSBs has dropped by more than 2% of energy while there has been a small increase in the rate of overweight and obesity over this time period.

## Competing interests

Flinders University was paid to do the secondary analysis of the Children's Nutrition Survey by the Beverage Council of Australia and Peter Clifton was paid to write the manuscript by Coca Cola South Pacific.

## Authors' contributions

LC, LM and LC did the original analysis of the survey and PC wrote the paper. All authors contributed to the manuscript and all authors read and approved the final manuscript.
